# Hypocritical blame is associated with reduced prosocial motivation

**DOI:** 10.1038/s41598-025-17698-4

**Published:** 2025-09-25

**Authors:** Luis Sebastian Contreras-Huerta, Hongbo Yu, Annayah M. B. Prosser, Patricia L. Lockwood, Molly J. Crockett, Matthew A. J. Apps

**Affiliations:** 1 Center for Social and Cognitive Neuroscience (CSCN), School of Psychology, Universidad Adolfo Ibáñez, Viña del Mar, Chile; 2 Department of Experimental Psychology, University of Oxford, Oxford, OX1 3PH U.K.; 3 Centre for Human Brain Health, School of Psychology, University of Birmingham, Birmingham, B15 2TT U.K.; 4 Center of Social Conflict and Cohesion Studies, Santiago, Chile; 5 Department of Psychological and Brain Sciences, University of California Santa Barbara, Santa Barbara, 93106 CA U.S.; 6 Marketing, Business and Society Division, School of Management, University of Bath, Bath, BA2 7AY U.K.; 7 Institute for Mental Health, School of Psychology, University of Birmingham, Birmingham, B15 2TT U.K.; 8 Christ Church, University of Oxford, Oxford, OX1 1DP U.K.; 9 Department of Psychology, Princeton University, Princeton, NJ 08540 U.S.; 10 University Center for Human Values, Princeton University, Princeton, U.S.

**Keywords:** Hypocritical blame, Prosocial motivation, Effort-based decision-making, Harm aversion, Moral behaviour, Psychology, Human behaviour

## Abstract

People often act hypocritically. One form of hypocrisy occurs when people blame others for transgressing moral principles they themselves have violated in the past. However, the psychological processes linked to this hypocritical blame are largely unknown. One possibility is that hypocritical blame is associated with the costs of being prosocial, such that a person could intend to help but is unwilling to put in the effort. Here, we test whether a measure of hypocritical blame that quantifies the discrepancy between willingness to profit from another’s harm, and blaming somebody else for similarly profiting, is related to the motivation to choose and then exert physical effort to benefit themselves or a stranger. Results revealed that hypocritical blame is associated with reduced prosocial motivation specifically, and not with how willing people are to exert effort for their own benefit. This effect was found in both a reduced willingness to choose to be prosocial and for energising prosocial acts. This suggests that the discrepancy between moral standards and actions is related to the willingness to overcome the costs of being prosocial, with some people being simply unwilling to exert the effort required to live up to their moral principles.

**Supplementary Information:**

The online version contains supplementary material available at 10.1038/s41598-025-17698-4.

## Introduction

Moral principles often guide how people act and are reinforced in society through the judgements we make of others. Judging someone as morally virtuous or vicious, depending how much they deviate from moral principles, serves to perpetuate these principles as norms. It has often been assumed that people guide both their moral decisions and their moral judgements according to the same standards. However, this is often not the case^1–5^. In fact, it is common that people show, at least to some degree, discrepancies between their actions and how harshly they judge others. For example, consider a politician who publicly condemns violations of Covid-19 restrictions as selfish, given their threat to public health, but then violates those same rules when it serves their personal interest, putting others at risk. There is a clear discrepancy between the politician’s moral judgements and their moral actions. This discrepancy has been identified by philosophers, psychologists, and the general public as a form of moral hypocrisy: hypocritical blame^1,4,6–9^.

Philosophical accounts have proposed that such hypocrisy could be due to having stricter moral standards for others, but more lax ones for ourselves^10,11^. Because of this double standard, hypocritical blamers are often seen as untrustworthy and are typically morally condemned^9,12–16^. However, a recent experimental study^9^ showed that underlying some manifestations of hypocritical blame there might be authentic moral principles. Participants made moral decisions about whether to inflict pain on another person in exchange for profit. A week after their decisions, and unbeknown to the participants, they judged how morally blameworthy similar decisions in the same task were. Hypocritical blame could then be estimated for each participant as the degree of discrepancy between their earlier decisions and their later moral judgments of similar behaviours. Using this measure, it was found that hypocritical blame was positively correlated with feelings of conflict and guilt during moral decision making. This suggests that, at least in some hypocritical blamers, failing to live up their moral standards might be linked to a weakness of the will (*akrasia*^16–18^.

One potential factor that might underlie a failure to live up to moral standards is the person’s sensitivity to the costs involved in acting morally, especially when those actions benefit others^19–21^. An important cost people have to incur in everyday life is effort, including when acting morally. Many prosocial actions—like helping a stranger to supporting a cause—require physical or cognitive exertion. Yet people are generally averse to effort, and this aversion is stronger for prosocial effort than for effort that benefits oneself^22–29^. Previous work suggests that if the costs to behave morally increase, people fail to behave according to their moral principles^19,21^. Thus, even though hypocritical blamers could genuinely share the moral principles they use to judge and blame others, aversion to the costs of performing helpful actions could overpower their good intentions^2,21,30^leading to a failure to live up to their own genuine moral standards^6,17–19^. Such an account would predict that people who show a high degree of hypocritical blame will be those who are more averse to exerting effort to benefit others. However, whether people’s levels of hypocritical blame are associated with people’s aversion to exerting effort for another’s benefit is unclear.

**Fig. 1:**
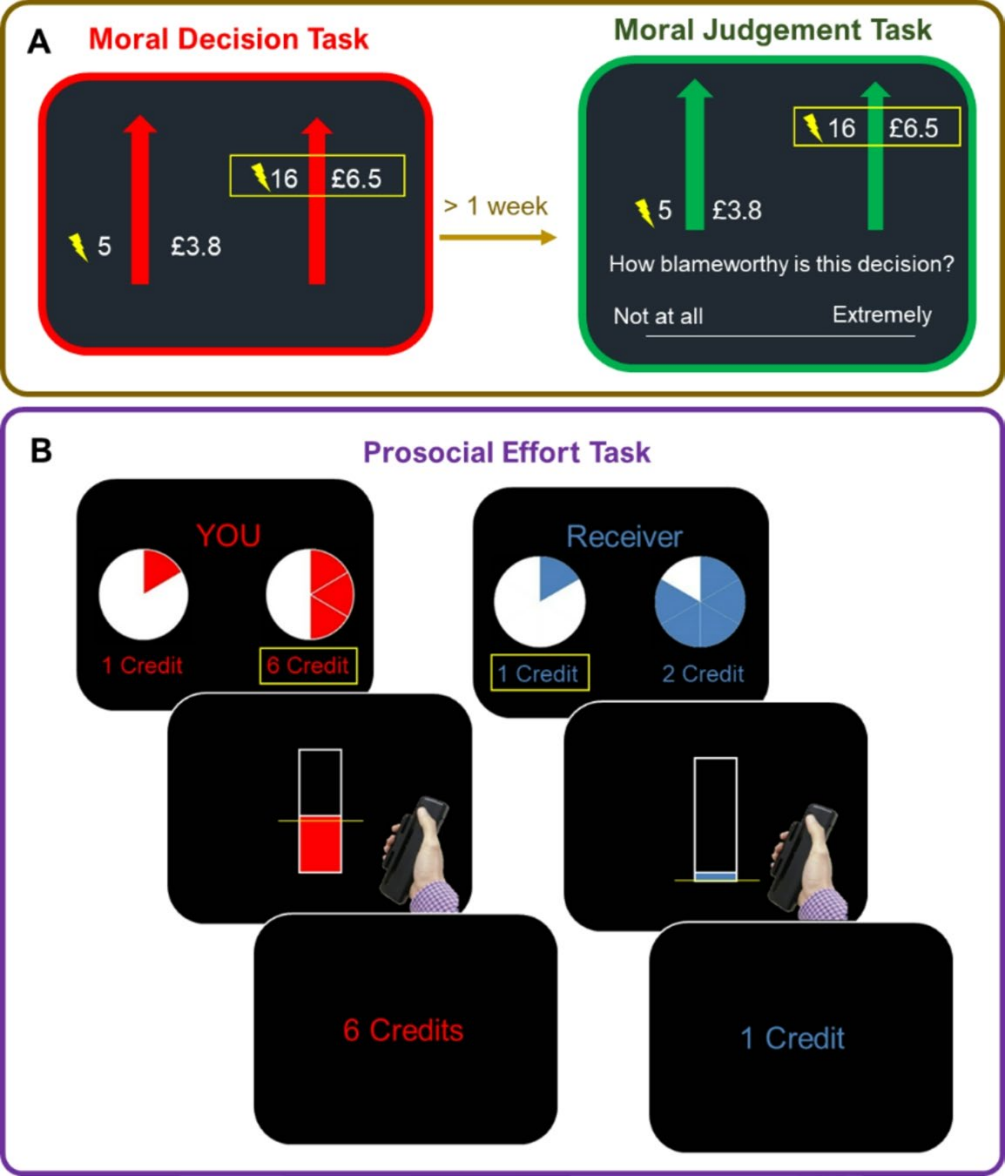
Behavioural measures. (**A)**. Hypocritical blame measure. Participants completed two tasks separated for at least a week - (i) a moral decision task, where participants traded-off profit against pain delivered to another person, choosing between a helpful (less profit and electric shocks) and a harmful option (more money and shocks), and (ii) a moral judgement task, where participants judged how blameworthy a series of decisions were when the harmful options were chosen. Unknown by participants, this set was the same trials they completed a week earlier. Hypocritical blame index was calculated using behaviour in these two tasks. (**B**). Prosocial motivation measure. In the prosocial effort task, participants chose between a rest, low reward option, and a work, higher reward offer. If the work option was chosen, the participant has to exert force thresholded to their own maximum voluntary contraction (MVC). For the work offer, different combinations of the five levels of effort (30–70% MVC) and reward (2–10 credits) were presented. Crucially, in half of the trials, rewards were delivered to the participant themselves, while in the other half it was given to an anonymous stranger.

Here, we test the association between hypocritical blame and motivation to effortfully help, We operationalised individual differences in hypocritical blame as the discrepancy between decisions in a moral task and the blame assigned on similar decisions made by others^9^ (Fig. 1A). Participants traded-off profit against electric shocks that were delivered to a stranger. Using computational modelling, we calculated the probability of each participant to harm others for profit based on their decisions in the task. At least a week later, participants completed a different task where they witnessed the same trials again, but this time they rated how blameworthy harmful decisions were. We calculated a hypocritical blame index as the product between probability of harm and their judgement towards the same actions, such that higher values indexed more judgement for actions that participants would perform themselves.

While self-report, trait-level prosociality captures individuals’ general preferences for benefiting others, many real-life prosocial acts require more than just the intention to help—they demand the willingness to overcome effort-related costs. Thus, measuring costly prosocial behaviour through effort-based paradigms provides a more ecologically valid index of real-life prosocial action. In our study, people’s willingness to exert effort to benefit others – prosocial motivation – was measured using a prosocial effort task, in which participants traded-off different levels of physical effort in exchange for different magnitudes of reward received by either participants themselves or another unknown person^25–27^ (Fig. 1B). Choosing to maximise rewards required participants to exert physical effort—squeezing a handle to a specific force level—which allowed us to quantify two distinct components of motivation: the decision to act prosocially despite effort costs, and the energisation of effortful actions once the decision was made.

Having these measures, two alternative hypotheses could be tested for how hypocritical blame is linked to both motivational aspects. Firstly, if hypocritical blame is linked to a reduced willingness to exert effort in general, rather than just for prosocial acts, it would be expected that people with high levels of hypocritical blame show less willingness to exert effort regardless of who the beneficiary may be, self or other. In contrast, if hypocritical blame is associated only with people’s willingness to act prosocially, then it will be associated with people’s aversion to effort only when another person benefits. These two hypotheses can be tested in terms of both people’s choices of whether to exert effort, and in terms of how much force – or energy – they exert into the effortful acts they have chosen to undertake.

We show that hypocritical blame is associated with effort aversion, specifically for prosocial acts. Those higher in hypocritical blame were less willing to choose to exert effort for another’s benefit, and exerted less force into those actions when they chose to perform them. These results suggest that hypocritical blame is closely linked to motivation.

## Results

### Hypocritical blame is highly prevalent and varies across participants

The hypocritical blame data and analysis are the same that appeared in Yu et al., 2022^9^. Participants (*n* = 61) first completed a harm aversion task measuring their moral decisions, where people traded-off money they can earn against electric shocks, choosing between a more harmful (but more profitable) option or a less harmful option^31,32^ (Fig. 1A). On half the trials, the harm –number of electric shocks – was delivered to oneself, and on the other half, to an anonymous person. This task allowed us to quantify the degree to which someone would themselves profit from harm being delivered to another person. Importantly, at least one week later, participants also completed a moral judgment task. In this task they witnessed the same options from the harm aversion task that they had performed before, but now they would rate how blameworthy those decisions were, between not at all to extremely blameworthy. Crucially, the harmful option was always chosen, and this task only had trials where shocks were delivered to someone else. Unbeknown by participants, this was the same set of trials they performed a week earlier. Thus, this task measured how much they blamed someone for choosing to profit from someone else’s harm.

**Fig. 2:**
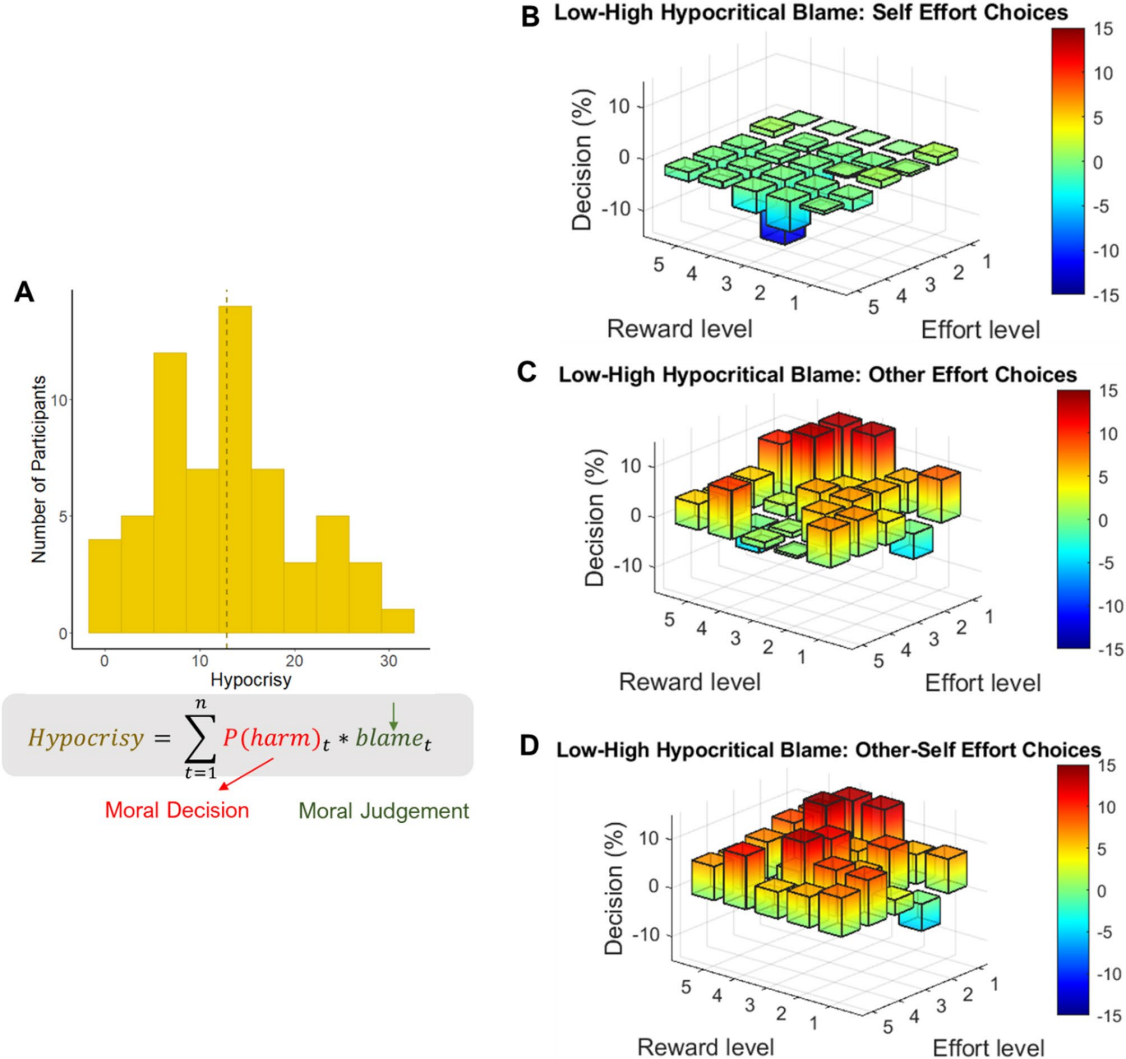
Hypocritical blame interacts with effort, reward and beneficiary in the prosocial effort task. **(A)**. Hypocritical blame was quantified by weighting the trial-by-trial moral blame ratings (from the moral judgement task) by the participant’s own probability to harm others (estimated from their prior moral decisions). The resulting hypocritical blame index followed a normal distribution. B-D. 3D surface plots illustrating differences in decisions to exert effort between participants with low versus high levels of hypocritical blame (median split used for visualisation only). Y -axis represents the percentage difference in choices to work (i.e., exert effort) between low and high hypocritical blame groups. Values range from − 15% to + 15%. Positive values indicate that participants with low hypocritical blame worked more often than those with high hypocritical blame, while negative values indicate the reverse. X- and Z-axes represent the different combinations between effort and reward levels. (**B)**. Self trials: No substantial differences between low and high hypocritical blame across effort and reward conditions. C. Other trials: Participants with low hypocritical blame were more likely to exert effort for others, particularly when high rewards could be obtained with low effort. D. Other – Self contrast: Participants with high levels of hypocritical blame showed a stronger bias to prioritise working for themselves over others, particularly when high rewards were obtainable with low effort. *Note: Median split was used only for visualisation. All statistical analyses treated hypocritical blame as a continuous variable.*

Using these tasks, a measure of hypocritical blame was quantified^9^ by comparing (i) the participants’ likelihood of making a harmful decision on the harm aversion task on the trials where the shocks were received by the other person (calculated through a computational model, see Materials and methods), with (ii) the blame they assigned for a harmful decision in the same trial. Hypocritical blame was defined by the sum of the blame assigned on each trial, weighted by the participants’ own likelihood to harm on the same trial. In this way, hypocritical blame was the discrepancy between the probability to harm in each trial and how harsh participants blame others for the same action. Thus, participants who assign a high level of blame on trials where they themselves are likely to choose the harmful option have a high hypocrisy score. In contrast, participants who almost never assign blame on the trials where they themselves are likely to choose the harmful option have a low hypocrisy score. With this operationalisation of hypocritical blame, 97% of participants displayed at least some level of hypocrisy, with a wide range of individual variation in the degree in which it is manifested, following a normal distribution (M ± SD = 12.9 ± 7.6; Kolmogorov-Smirnov normality test, Fig. 2A).

### Hypocritical blame is linked to prosocial motivation

In the same experimental session where the moral judgement task was performed, participants completed the prosocial effort task, where they decided between a low reward, rest option, and a variable high reward, high effort option (five levels for effort and reward respectively, Fig. 1B). Importantly, on half of the trials the reward was for participants themselves, while in the other half the money was received by an anonymous receiver. This person was a different receiver relative to the harm aversion task, to avoid the potential influence of reciprocity (see Material and Methods and Supplementary Methods for details). Using this task, we tested whether hypocritical blame was linked to motivation to work to benefit others and/or self.

We used a linear mixed effects model (MM) predicting decisions to work versus rest with predictors of level of effort, magnitude of reward, and beneficiary of the reward, together with their potential interactions. In doing so we could examine whether it was specifically effort, rather than reward, that was associated with hypocritical blame. These models had a random intercept for each subject, and random effects for effort and reward levels, as previous studies have shown that sensitivity to this information vary across participants^25,29^. First, we tested whether adding hypocritical blame as a variable into this MM improved model fitting, demonstrating a better prediction of choices to exert effort by including the blame measure. We found that a model containing effort, reward, beneficiary and hypocritical blame outperformed a model without the latter variable (AIC: simple model = 5457.7; hypocrisy model = 5446.8, Supplementary Fig. S1). A loglikelihood ratio test confirmed that the hypocrisy model significantly improved the model fit (χ^2^_diff_ = 28.93, df_diff_ = 9, *p* < 0.001) suggesting that hypocritical blame is important to predict decisions to exert effort for reward.

Within the winning model (for detailed results see Supplementary Table S1), a four-way interaction was found between hypocritical blame, effort, reward and beneficiary (b = 0.27, SEM = 0.1, z = 2.7, *p* < 0.007). If hypocritical blame specifically modulates prosocial effort, its influence should differ depending on whether the effort benefits oneself or another. To explore this interaction, we conducted a series of post-hoc analyses comparing the slopes of hypocritical blame predicting decisions to exert effort across reward–effort combinations, separately for self and other trials. Differences between self and other emerged primarily at lower effort levels and moderate-to-high rewards (see Supplementary Table S2 and Supplementary Figure S2). Notably, as effort increased, the difference between self and other trials diminished and became non-significant. Similarly, no significant differences were observed at the lowest reward level, regardless of effort. This pattern is visualised in Fig. 2B–D, which displays the percentage of trials in which participants chose to exert effort, broken down by reward, effort, beneficiary, and levels of hypocritical blame (median split). The results show that individuals high in hypocritical blame were less willing to exert effort to benefit others—especially when helping was relatively easy and highly rewarding—while their willingness to work for self-benefit remained largely unchanged. This suggests that hypocritical blame is associated with reduced sensitivity to others’ gains and heightened sensitivity to effort costs in prosocial contexts.

**Fig. 3:**
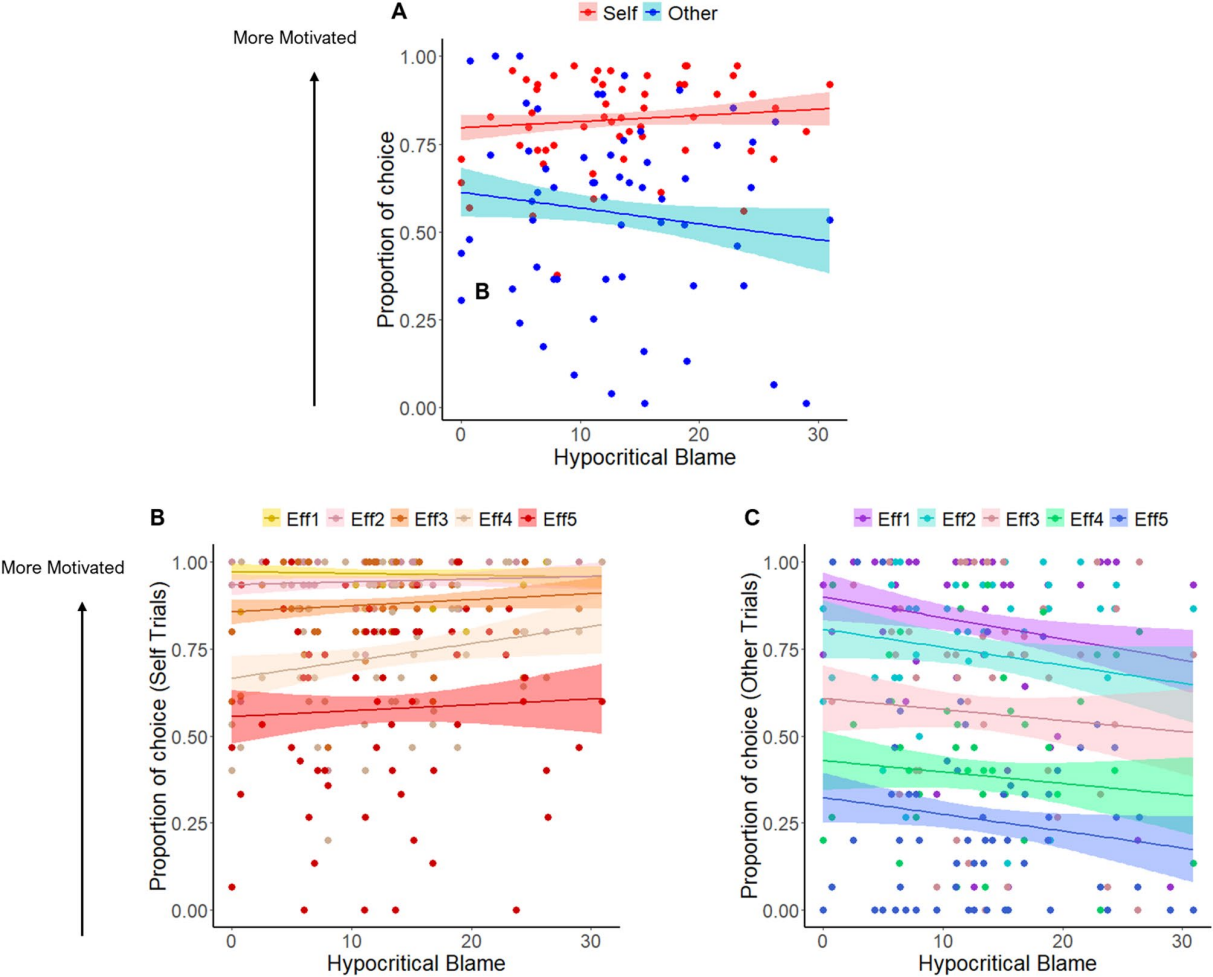
Hypocritical blame is associated with less willingness to work for others compared to self. (**A)**. Y-axis shows proportion of choosing the work versus the rest option. Participants get less motivated to work for others compared to self as they have higher scores in hypocritical blame (**B-C**). Effects of hypocritical blame on choices according to effort and beneficiary. Hypocritical blame is associated with less motivation for others across effort levels (**C**) compared to self (**B**). Shaded areas show the 70% confidence interval around the slopes. Individual points show the score of each participant for each condition. Eff = Effort Level.

Consistent with the above results, hypocritical blame also showed a significant three-way interaction with beneficiary and effort (b = 0.27, SEM = 0.11, z = 2.5, *p* < 0.02) and a two-way interaction with beneficiary (b = −0.52, SEM = 0.11, z = −4.54, *p* < 0.001), but not a main effect by its own. These results support the four-way interaction described above - as participants scored higher in hypocritical blame, they worked less for others compared with self (Fig. 3). Finally, we found similar effects revealed by previous studies showing that participants were less willingness to work for others than for self, especially in high effort trials, and in trials with low rewards on offer (see Supplementary Table S1).

Finally, an alternative hypothesis is that the observed effects of hypocritical blame on prosocial effort reflect a domain-general prosocial disposition. To test this, we examined whether prosocial tendencies in the harm-aversion task, as estimated by our computational model (κ_other_; see Materials and Methods), correlated with prosocial effort tendencies in the effort task (λ_other_; model described in Supplementary Methods, see descriptive statistics of the parameters in Supplementary Table S3). Because κ_other_ was already included as a regressor of no interest in our models, this analysis provides a complementary test of overlap across tasks. The correlation was not significant (Spearman ρ = 0.10, *p* = 0.46), suggesting that harm aversion alone does not explain decisions toward others in the prosocial effort task. For completeness, we also examined correlations for decisions in the self condition (κ_self_ and λ_self_) and for self–other differences, which are reported in Supplementary Table S4. Together, these analyses indicate that hypocritical blame, rather than a domain-general prosocial disposition, best accounts for prosocial effort decisions.

### Hypocritical blame is associated with reduced energisation of actions when working for others

#### Higher levels of hypocritical blame are associated with reduced force exertion when invigorating actions that benefit others

Even after people make a decision to exert effort, they might not always put as much energy into those actions for others compared to self. Then, we tested whether hypocritical blame was linked to how much force participants exerted into actions when helping others. We examined the “normalised force” for each trial in the prosocial effort task – calculated as the area under the curve (AUC) of the force normalised to a participants maximum AUC across all trials. We first tested whether including hypocritical blame into the model improved model fitting. Similar to what we found looking at decisions to work, a model that included hypocritical blame outperformed a simpler model with only effort, reward, beneficiary and their interactions as independent variables (AIC, simple model = −17179, hypocrisy model = −17193). This was later confirmed by a loglikelihood test that revealed a significant model fitting improvement by the hypocrisy model (χ^2^_diff_ = 27.59, df_diff_ = 7, *p* < 0.001).

**Fig. 4:**
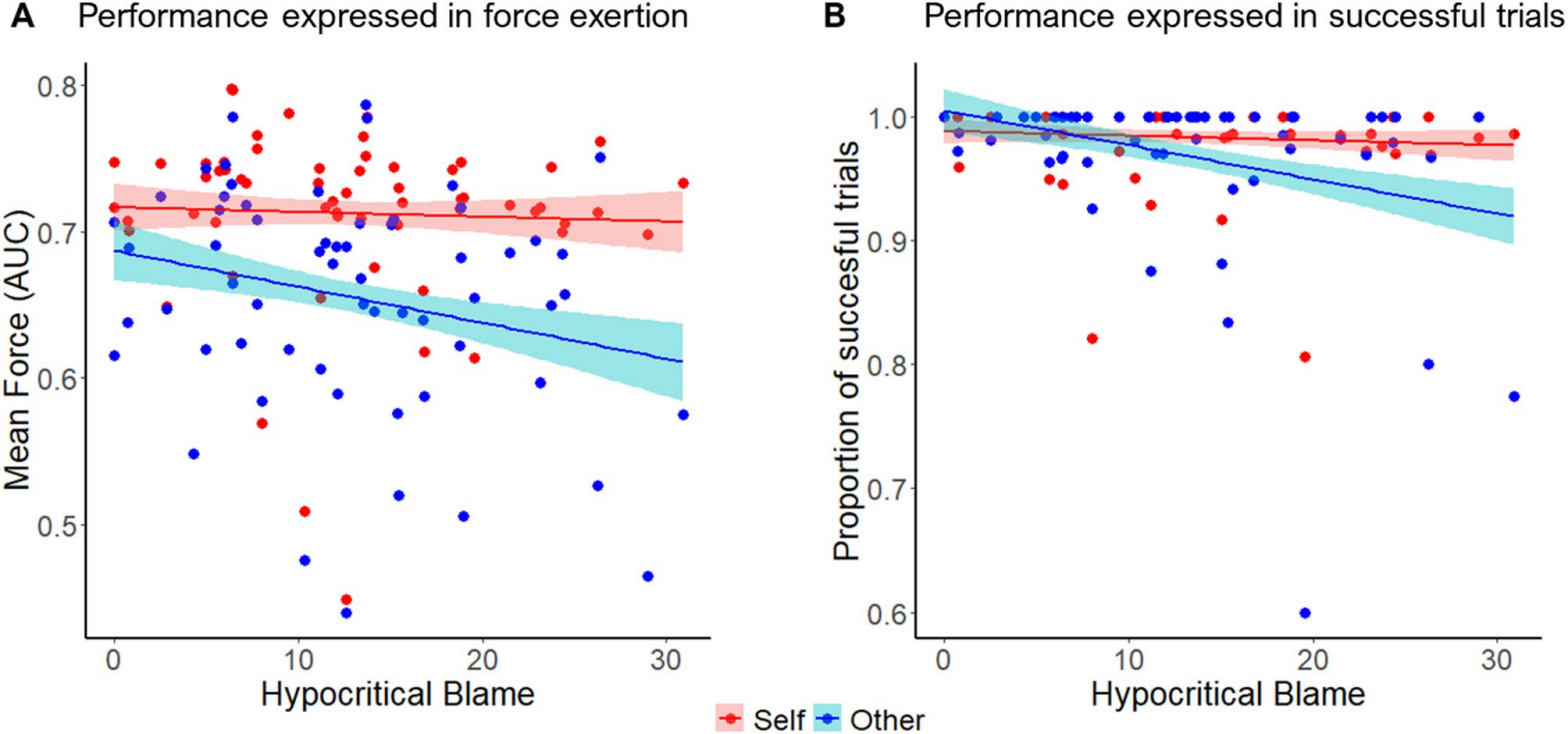
Hypocritical blame is linked to lower performance in actions that benefit others compared to self. (**A**). Hypocritical blame is associated with less force exerted for others compared with self. Participants who scored high in hypocritical blame showed more differences in how they exerted force for others compared to self when similar effort was required, suggesting more superficiality in their prosocial actions. Y-axis depicts the mean area under the curve (AUC) during the 3 s force period normalised to participants maximum level of force exerted across trials. (**B**). Hypocritical blame is associated with less success in trials benefiting others. As people score higher in hypocritical blame, they fail more in trials where the benefit is received by others compared with themselves. Y-axis depicts the proportion of successful vs. failed trials. Shaded areas show the 70% confidence interval around the slopes. Individual points show the score of each participant for each condition.

An interaction between hypocritical blame and beneficiary was found (b = −0.01, SEM = 0.002, t = −3.94, *p* < 0.001), indicating that as participants are more hypocritical, the difference in force exertion between self and other increased (Fig. 4A), such that they exerted less force for actions that benefitted others relative to self than participants low in hypocritical blame. Thus, the discrepancy between moral judgements and actions is associated with higher gaps between self and other action invigoration. Furthermore, this model revealed main effects of effort (high effort levels require more force exertion), reward (participants exerted more force when the reward was high), and beneficiary, together with an effort x beneficiary interaction, showing that participants generally exerted less force for others than for self, especially in high effort trials, replicating previous studies (see Supplementary Table S5, and Supplementary Figure S3).

Since participants, regardless of hypocritical blame, chose to benefit others much more when the effort required was low compared to high, the above effect could be accounted by a reduced sampling of trials at the higher level of effort in the other condition. That is, there are less high effort trials for other in people who are highly hypocritical. To confirm this was not driving the effect, we examined if removing the higher effort trials still revealed an effect of hypocritical blame. Consequently, we removed consecutively the three highest effort levels from the model testing each time whether the effect was still present. The hypocritical blame x beneficiary interaction proved to be a robust effect and it was present even if the highest effort level (70% of maximum voluntary contraction, MVC; t = −4.1, *p* < 0.001), as well as the two highest effort level (60–70% MVC; t = −2.75, *p* < 0.006), and the three highest effort levels (50–70% MVC; t = −2.17, *p* < 0.04) were removed. Thus, the people who were highest in hypocritical blame simply exerted less force for others, even when they had chosen to do so.

#### Hypocritical blame is linked to higher failure to exert the required force when performing prosocial actions

As effort levels are set as a percentage of participants’ MVC in the prosocial effort task, all effort levels should be attainable, and failure indicates a reduction in the motivation to exert the required effort even when they freely chose to undertake it. In fact, success rates were very high overall in the prosocial effort task. Participants on average obtained the reward on offer in 98.3% of the self trials (SEM = 0.5%), and in 96.9% of the other trials (SEM = 0.8%). Is hypocritical blame related to failure to succeed at the required effort level? The above results on force exertion suggests that this could be the case. In order to test whether hypocritical blame modulated the probability to succeed in a given trial, two different models were built using the same approach described above, i.e. a simple model against a hypocrisy model, but now predicting the binary variable ‘success’. Note, however, that the results revealed in this analysis could be driven only by a number of participants in only a number of trials due to the high success rates of this task. The following results should be therefore interpreted with caution.

The hypocrisy model improved model fitting relative to the simple model according to the AIC (simple model = 953.7, hypocrisy model = 949.1). A loglikelihood test confirmed that the hypocrisy model significantly outperformed the simple model (χ^2^_diff_ = 18.61, df_diff_ = 7, *p* < 0.01). Crucially, this model revealed that hypocritical blame interacted with beneficiary (b = −0.74, SEM = 0.3, z = −2.48, *p* < 0.02, Fig. 4B), such that people who scored high in hypocritical blame failed more in other trials compared with people who scored low, showing bigger differences in success between self and other. These results indicate that participants with high scores in hypocritical blame not only exerted less force for others than for themselves for similar actions, but also, they did so to the extent that more often failed to energise the required force to obtain the reward on offer. Finally, the hypocrisy model revealed main effects of effort, indicating that people failed more as trials get harder, and beneficiary, showing that people failed more in other compared to self trials (see Supplementary Table S6).

Given that hypocritical blame modulated success in other trials, it could be argued that its effect on force exertion might have been driven only by those trials where participants exerted such a low amount of force that they did not achieve the required effort goal. However, this seems unlikely, since the effect of hypocritical blame on force remained even if highest effort trials (50–70% of participants’ MVC), which are more likely for participants to fail, are removed from the model. Despite this, and to add robustness to these results, we performed a final analysis to test the effects of hypocritical blame on normalised force building again a simple and a hypocrisy model on trial-by-trial force exertion but this time considering only trials where participants succeeded. Again, the hypocrisy model significantly improved model fitting relative to the simple one (AIC: simple model = −18858, hypocrisy model = −18865, χ^2^_diff_ = 20.39, df_diff_ = 7, *p* < 0.005). Crucially, the hypocritical blame x beneficiary interaction was maintained (b = −0.003, SEM = 0.001, t = −2.18, *p* < 0.03), indicating that people who scored high in hypocritical blame exerted less force for others across trials. A three-way interaction between hypocritical blame, beneficiary and effort was also significant in this model with only successful trials (b = 0.004, SEM = 0.001, t = 2.64, *p* < 0.01), suggesting that the modulation of hypocritical blame on the force exerted for self and other varied across different effort levels. This effect is likely to be triggered by the removal of the failed trials from the analysis, which corresponds to the manifestation of low motivation in force exertion especially at the higher effort levels. Thus, while hypocritical blame effects on lower effort levels remained in this model, its effects in the harder ones were diminished by eliminating these failed trials.

## Discussion

Every day people blame others for actions that they judge as morally wrong, denoting in this way their own moral standards. Yet, individuals frequently fail to meet those same standards themselves. In this study, we tested the hypothesis that such hypocritical blame is linked to reduced motivation to overcome the personal costs of acting morally—especially in prosocial behaviours that benefit others^19^. Using decision-making tasks assessing hypocritical blame and prosocial effort motivation within the same participants, we found that people with higher levels of hypocritical blame, i.e. wider gaps between moral judgements and actions, (i) were less willing to put in effort to benefit others relative to self, and (ii) exerted less force when performing effortful actions to benefit others compared to their own. These findings suggest that higher hypocritical blame is associated with diminished motivation to incur the costs of prosocial action, which may contribute to failures in living up to one’s own moral standards.

This study revealed that hypocritical blame is associated with less willingness to incur effort costs specifically for prosocial actions, but not for self-benefitting acts. This aligns with the fact that the motivation to put in effort for ourselves may be fundamentally different that choosing to help others, which may be more of a moral choice. Choosing whether to exert effort to obtain rewards is a goal-directed behavioural problem that even non-social animals must consider. People vary in their willingness to work in the absence of any social context. However, prosocial effort is morally relevant. Doing something to benefit someone else requires a personal cost^33^ without an immediate or well defined benefit to oneself. People high in hypocritical blame may be quick to judge others’ actions, as judging is not costly. However, when action is required—particularly when the benefits accrue to others—they may struggle to overcome personal costs. This may reflect different sensitivities to self versus other interests in moral hypocrites^34,35^. This is consistent with evidence suggesting that low hypocritical people might be more self-driven by the welfare of others, valuing self and other benefits more equally^35^in contrast with people who act morally only to conform to the social norm^10,36,37^. Indeed, previous work has shown that people scoring high on moral responsibility are not necessarily less hypocritical, but those who score high on self-motivated moral intentions display more alignment between moral principles and actions^2,10,35–38^. Our results suggest that this gap might be linked to differences in effort sensitivity and reward valuation when actions benefit others. Thus, while judging others is easy, helping others is costly—and hypocritical individuals may fail to live up to their own standards due to this asymmetry.

Strikingly, participants with high levels of hypocritical blame were less willing to work prosocially especially in trials where the effort cost was low to moderate and the benefit high to moderate. At first, this result seems counterintuitive, since it is in the high effort levels where people in general show more reluctance to incur in prosocial effort^25–29^and hypocritical blame could have just augmented this effect. However, people have consistently shown high motivation to work in easy trials regardless of the beneficiary, a ceiling effect that could impact on the current results. Thus, as hypocritical blame was linked to low motivation in these trials, the difference in this spectrum of the design is more pronounced compared with participants low in hypocritical blame. In fact, we did not see effects of hypocritical blame on decisions in the highest effort levels between self and other - people were equally demotivated to work for others regardless of hypocrisy. Furthermore, the effects of hypocritical blame were evident at higher levels of rewards, especially in combination with low effort costs. Taken together, these results suggest that the effort sensitivity of hypocritical people is manifested in social situations where most people would be willing to work to benefit others. Hypocritical blamers might reduce self-costs, even if those costs are small, especially in contexts where there is not a clear moral norm for behaviour like the prosocial effort task - i.e. not putting in effort to help others is not as socially prohibitive as harming others for profit^21,39–42^.

Intriguingly, hypocritical blame in the current study was not only associated with less willingness to choose to work for others, but also, when they decided to do so, they energised less the prosocial actions compared with self-benefiting behaviour ones. Indeed, sometimes people high in hypocritical blame failed to achieve the required force to obtain the money for others due to their poorer performance. These results suggest that hypocritical blamers’ interest towards others could also be *superficial* – they may appear prosocial in their willingness to help on the surface, but the effort they invest to benefit others suggests otherwise. Consequently, hypocrites might display moral principles mainly through their judgements and desires, as these are not costly, but not through actual actions. When faced with the opportunity to avoid personal cost, they would do so—while still maintaining the appearance of moral integrity^2,38^.

Research on goal-directed behaviour can offer an explanatory framework for interpreting these results. In reward processing, two components can be identified^43,44^ - a hedonist impact of pleasure given by the reward, a ‘*liking’* component; and the incentive saliency of the reward, a ‘*wanting’* component, that drives the agent towards a reward-seeking behaviour that leads to a willingness to exert effort. Even though these two components are related to each other, they can, in principle, be dissociated and linked to distinct neural mechanisms. The current results could be interpreted as hypocritical blamers ‘liking’ the idea of being moral – they share moral principles that lead to high moral standard reflected in their judgements and desires, but they fail in ‘wanting’ to be moral, and thus do not overcome self-costs to benefit others.

The present results, especially the superficiality of the hypocritical prosocial decisions, could be interpreted as supporting a claim that hypocritical blame might be a deceptive behaviour. That is, people might want to display moral standards that they have no intention of meeting themselves. However, this does not necessarily have to be the case^6,17,18^. First, participants, regardless of hypocritical blame, decided to help others in a high number of trials, including ones with high effort costs. Second, in the vast majority of the trials, participants, regardless of hypocritical blame, achieved the force required to obtain the reward on offer. Finally, failing to meet their prosocial and moral standards might trigger guilt and frustration in the hypocritical blamer due to their moral failures^6,17,18,45,46^. Indeed, according to previous results^9^hypocritical blamers might feel conflict and guilt when they fail to behave according to their moral principles. Taken together, therefore, it seems more appropriate to consider hypocritical blame as the extreme of a continuum, associated with reduced motivation to overcome self-costs to benefit others. People with higher levels of hypocritical blame still worked for others, only that they did so to a lesser degree than the lower extreme, as they are more sensitive to their self-interest when benefitting others.

Importantly, our results underscore the value of using behavioural tasks that involve real costs to the participant when studying prosocial behaviour. Unlike self-report measures, which are often influenced by social desirability biases or limited introspective access, our tasks capture concrete decisions that benefit others—or the self—at a personal cost. This feature allowed us to identify a key hallmark of hypocrisy: the discrepancy between moral intentions and actual behaviour. We propose that hypocritical blame may reflect a broader tendency to endorse moral principles through judgment or stated intentions, yet fail to act accordingly when moral behaviour becomes effortful or personally costly. Capturing these costs is therefore essential to reveal the motivational foundations of hypocritical behaviour.

One alternative explanation, however, is that the observed relationship between hypocritical blame and prosocial effort reflects a broader, domain-general prosocial disposition. Indeed, in a previous study^28^we found that individuals who were more prosocial in a harm-aversion task also tended to be more prosocial in an effort-based task, suggesting some shared motivational component. However, it is unlikely that our current results are explained solely by this link. First, all our models included the harm-aversion parameter as a covariate, allowing us to account for variance associated specifically with that construct (see Material and Methods). Second, in our previous study^28^the strongest relationship between tasks was not for behaviours that benefit others per se, but for choices that benefit others relative to the self. In contrast, hypocritical blame focuses exclusively on behaviour toward others, not on relative trade-offs. Indeed, we did not find evidence of a correlation between harm aversion for others and willingness to work for others in our present study. Finally, the link between hypocritical blame and prosocial effort emerged not only at the level of decisions but also in actual task performance. Participants high in hypocritical blame did sometimes express prosocial intentions, but showed poorer execution when prosocial actions required personal effort. This pattern aligns conceptually with our motivational framework of hypocrisy, which emphasises a disconnect between moral endorsement and motivated behaviour. Taken together, these findings suggest that the association between hypocritical blame and prosocial effort cannot be fully explained by general prosocial tendencies or by harm aversion alone. Instead, they point to a specific motivational deficit: individuals prone to hypocritical blame may endorse moral standards when doing so is easy or cost-free, yet lack the motivation to act prosocially when such actions entail personal cost.

Future research could build on this work by addressing some limitations of the current study. First, it could test directly whether moral hypocrisy is associated with deceptive or genuine moral intentions. Second, it could extend our findings to scenarios where the relevant cost is not effort, where hypocrisy takes other forms (e.g., discrepancies between attitudes and actions, or judgments for self vs. others), and where judgments and actions are made publicly versus privately. Third, it could also examine hypocrisy in more ecologically valid contexts that are emotionally charged, socially embedded, and reputation-relevant^38,47,48^— for example, condemning others for failing to recycle while engaging in carbon-intensive travel, or publicly judging cheating while behaving dishonestly in private. In contrast, our paradigm distilled hypocrisy into a controlled laboratory setting involving the moral norm of not harming others for personal gain. While this abstraction facilitates precise measurement, it lacks the ‘hot’ passion and societal salience that typically accompany hypocrisy in daily life. Embedding hypocrisy paradigms in richer social contexts would therefore help bridge the gap between controlled experimental designs and the socially impactful hypocrisy that shapes moral discourse. Our findings nonetheless suggest that even in a stripped-down setting, individuals who show greater hypocritical blame are less willing to exert prosocial effort, pointing to a motivational mechanism—heightened cost sensitivity—that may generalise across contexts. From this perspective, future research could also shed light on what personality traits modulate the motivational aspects of hypocritical blame^2,11,35,36,46,49–51^. Taken together, the present results open a research opportunity for future investigations to illuminate the psychological aspects of moral hypocrisy.

Finally, our measure of moral hypocrisy was derived from two tasks administered one week apart: the moral decision task always came first, followed by the moral judgment task. This fixed order could be seen as a potential limitation, as participants who remembered specific trials might have their judgments influenced by that memory. However, we believe our design helps avoid several important confounds. First, prior work shows that observing others’ moral decisions can influence one’s own decisions^52^; placing the judgment task before the decision task could therefore have primed participants’ choices. Second, it is unlikely that participants vividly remembered specific trials from the first session. The tasks were separated by time, interspersed with unrelated activities. Moreover, the trials included a wide range of shock–money combinations, further reducing the likelihood that participants could recall exact scenarios. Crucially, and even if such recall occurred, our hypocritical blame index was not based on direct trial-by-trial comparisons. Instead, it captured the discrepancy between participants’ explicit blame judgments and the model-derived probability of them acting immorally in the same situation, based on their own behavioural patterns. This approach reflects stable moral tendencies rather than episodic memory of specific acts, and highlights the value of using computational modelling in prosocial and moral behaviour research. Therefore, it is unlikely that our task order introduced a systematic bias in moral judgments. Nonetheless, future studies should consider including explicit memory or narrative-based measures to further explore the role of memory, self-awareness, and narrative processing in moral evaluation^53^.

In summary, here we tested whether hypocritical blame, a discrepancy between moral judgements and actions, is associated with lower prosocial motivation in the form of a reduced willingness to exert effort for others’ benefit. Our results highlight that those that hypocritically blame others, may do so because they are too sensitive to the costs to be moral. These results open an investigative door into a motivational, goal-directed component of moral hypocrisy.

## Materials and methods

### Participants

All protocols were approved by the ethics committee of the University of Oxford (R50262/RE001), and followed the Helsinki Declaration of 1964. All participants gave written, informed consent at the beginning of the study. The participants used in this study were part of the sample used by Yu et al., 2022^9^ (*n* = 62). One of these 62 participants never chose to help others in the prosocial effort task, meaning an absence of sufficient variance of force data, crucial for hypothesis testing. Therefore, 61 participants were included in the analyses reported here (age M = 22.6, SD = 3.8, 34 females). All participants gave written informed consent and were financially compensated for their time.

### General procedure

Participants took part of a multi-stage, multi-task study researching social decision-making. As part of this study, participants had two experimental sessions. First, participants completed the harm aversion task. Second, and at least one week later (range = 7–74 days, median = 13 days), participants attended to a behavioural session, where they completed the moral judgement task and the prosocial effort task (see Yu et al., 2022^9^ for details about the general procedure).

### First experimental session

#### Harm aversion task

Before completing the harm aversion task, participants completed a pain thresholding procedure^31,32,52^ aiming to familiarise them with the painful shocks to be traded-off in the decision task, and to identify the physical intensity according to each participant’s subjective pain scale and match it across the sample. Next, participants went through a role assignment procedure where they were mock-assigned the role of Deciders while a confederate was assigned the role of Receiver, following a well-established protocol^25,31^ (see Supplementary Methods).

After these procedures, participants received instructions about the task and completed some practice trials. They were told that one trial was going to be randomly selected and implemented at the end of the session, and they were ensured that their decisions would be completely anonymous and confidential. Participants then completed the harm aversion task, measuring their moral behaviour^31,32,52^. In this task, participants traded-off a certain number of electric shocks against financial profit. Crucially, in half of the trials participants believed that the electric shocks were delivered to another unknown person, the Receiver, while in the other half shocks were delivered to participants themselves, the Deciders. Money, on the other hand, was always obtained by the participants/Deciders.

In every trial, participants decided between two options: a harmful option, associated with more shocks for more profit, and a helpful option, associated with less shocks but in exchange of less money. 72 trials were included per condition (generated using the criteria described in Crockett et al., 2017^32^), plus four catch up trials (harmful options were associated with lower profit), making a total of 76 trials. In half of these trials, the harmful option was on the left side of the screen, while in the other half they were on the right. These 76 trials were duplicated to have the same trials for self and other conditions. These trials were displayed in two blocks of 76 trials to each participant, with each block equal amount of self and other trials. Four sets of 76 trials were produced following this protocol, and participants were assigned randomly one of these sets. This trial generation protocol ensured that number of shocks were decorrelated from the amount of profit (|r| < 0.07, *p* > 0.53).

After the harm aversion task, one trial was randomly selected and implemented. Thus, if a self trial was selected, shocks and money corresponding to participants’ choice in that trial were delivered to them at the end of the session. However, if an other trial was selected, participants received the money and were told that shocks will be delivered to the Receiver at the end of their session. Finally, participants answered a few debrief questions about the study.

### Second experimental session

#### Moral judgement task

To operationalise hypocritical blame, decisions in the harm aversion task had to be contrasted with moral judgements for similar actions. Thus, the moral judgement task aimed to examine how much people rate actions that they have previously performed as blameworthy when they are performed by others^9^. At least one week after participants completed the harm aversion task, they had an experimental session where they undertook the moral judgment task. In this task, participants were presented with a subset of the trials that they completed in the harm aversion task, i.e., all the trials in the other condition where the money was for the Decider and the shocks were for the Receiver (72 trials). On each trial, the harmful option was chosen. Participants were asked to judge the blameworthiness of each harmful choice on a visual analogue scale ranging from 0 (not at all blameworthy) to 100 (extremely blameworthy). Using this task, we calculated each participant’s degree of hypocritical blame, which quantifies discrepancies between their blame judgments and the choices they made a week earlier (see below).

#### Prosocial effort task

Participants completed the prosocial effort task^25–27^ after the moral judgement task in the same behavioural session. They were told that they would continue their role as Deciders, while a Receiver would be paired with them from the pool of Receivers in the study. In the prosocial effort task, participants traded-off physical effort for monetary rewards in the form of credits. Crucially, in half of the trials the money was received by the Receiver, while in the other half profit was obtained by the participants themselves, the Deciders. In every trial, participants chose between two options: a baseline option, associated with no exertion of effort (i.e. 3 s of rest) for a low reward level of one credit, and a work offer, associated with higher amounts of reward (2–10 credits) for variable levels of effort exertion (30–70% of their maximum voluntary contraction) to obtain them. Once participants made their choice, they were required to perform the specific effort level squeezing a handle with their dominant hand for at least one second in a three second window. Failing to do so meant to get zero rewards for that trial. Each combination of effort and reward were repeated three times per condition, having 75 trials per beneficiary. This task allows to measure two important aspects of motivation: prosocial effort-based decisions, and the energisation of actions for self and other. For the latter, two indexes were used: (i) the force exerted for each trial where participants decided to work, and (ii) participants’ success in accomplishing the required level of physical effort to obtain the reward on offer. In principle, there should not be differences between self and other as participants have the alternative of resting for a low reward, and as each effort level is adjusted to a percentage of each participant’s maximum voluntary contraction (MVC), which ensure a high level of success. Thus, if participants choose to work for a specific amount of effort level, they should exert, on average, the same amount of force in that effort level regardless of whether the reward is for themselves or not. However, participants in fact generally exert less force into the same actions when they benefit others than self^25,26^.

Prior making decisions in the main task, participants’ MVC was determined, when they squeezed the handle as strongly as they could. This ensured effort levels were idiosyncratically set for different participant’s grip strength. After this, participants experienced each effort level three times, getting familiar with effort levels and their display on the screen. Once these two processes were completed, participants performed the prosocial effort task.

### Analysis of the behavioural data

#### Calculating hypocritical blame

To compute the degree of hypocritical blame in participants, we determined the discrepancy between participant’s own aversion to harming others and the level of blame that they attributed to similar actions, following the methods used in Yu et al., 2022^9^. Given that we assessed participants’ real choices in the harm aversion task, we could contrast these choices with the moral judgments they rendered towards the decisions made the same set of trials. We thus quantified each participant’s degree of hypocritical blame by comparing (i) their probability of choosing he harmful choice in each trial of the harm aversion task and (ii) the blame they assigned on each trial when a harmful decision was made in the moral judgment task. We used a computational model that has been extensively validated in previous work^31,32,54^ to calculate participants’ likelihood to harm in each trial. Here, the probability to harm is the softmax transformation of the subjective value of the harmful relative to the helpful option, ΔV. Thus, for participant *j* and trial *i*:

1 $$\:{{\Delta\:}V}_{j\left(i\right)}=\left(1-{\kappa\:}_{j}\right)\:{{\Delta\:}m}_{j\left(i\right)}-\:{\kappa\:}_{j}\:{{\Delta\:}s}_{j\left(i\right)}$$

2 $$\:{P\left(harm\right)}_{j\left(i\right)}=\:\frac{1}{1+\:{exp}^{{-\beta\:}_{j}\:{\varDelta\:V}_{j\left(i\right)}}\:}\:\left(1-2{\epsilon\:}_{j}\right)+\:{\epsilon\:}_{j}$$

Where *Δm* and *Δs* are the difference in money and shocks respectively between the harmful and the helpful options. The free parameter *κ* represents how sensitive participants are to shocks over money. Importantly, κ has different values for self and other trials (κ_self_ and κ_other_, respectively). We transformed ΔV into probabilities using a softmax function, where *β* was a participant-specific inverse temperature parameter indicating the stochasticity of the decisional process. A lapse rate parameter *ε* was also included which captured task-irrelevant noise.

To compute hypocritical blame, we used choices made only in the other trials. Hypocritical blame was defined as the sum of the judgement made by the participant in each trial weighted by participants’ probability to harm in that trial, such that:

3 $$\:{Hypocritical\:Blame}_{j}=\sum\:_{i}{blame}_{j\left(i\right)}\text{*}{P\left(harm\right)}_{j\left(i\right)}$$

Where *blame*_*j(i)*_ is participant j’s blame on trial i of the moral judgment task. *P(harm)*_*j(i)*_ is the probability to harm of that participant in the same trial extracted from their performance in the harm aversion task.

### Testing the relationship between hypocritical blame and prosocial motivation

For decisions, we tested whether hypocritical blame was associated with prosocial motivation with two mixed models (*glmer* function in R):

Simple model

4 $$\:{DW}_{i}=\:{\beta\:}_{0j\left[i\right]}+\:{\beta\:}_{1j\left[i\right]}{R}_{i}+{\beta\:}_{2j\left[i\right]}{E}_{i}+{\beta\:}_{3}{B}_{i}+\:{\beta\:}_{4}{R}_{i}{B}_{i}+\:{\beta\:}_{5}{E}_{i}{B}_{i}+\:{\beta\:}_{6}{E}_{i}{R}_{i}+\:{\beta\:}_{7}{E}_{i}{R}_{i}{B}_{i}$$

Hypocrisy model

$$\:{DW}_{i}=\:{\beta\:}_{0j\left[i\right]}+\:{\beta\:}_{1j\left[i\right]}{R}_{i}+{\beta\:}_{2j\left[i\right]}{E}_{i}+{\beta\:}_{3}{B}_{i}+{\beta\:}_{4}H+{\beta\:}_{5}{R}_{i}{E}_{i}+\:{\beta\:}_{6}{R}_{i}{B}_{i}+\:{\beta\:}_{7}{R}_{i}H+\:{\beta\:}_{8}{E}_{i}{B}_{i}+\:$$

5 $${\beta\:}_{9}{E}_{i}H+\:{\beta\:}_{10}{B}_{i}H+\:{\beta\:}_{11}{{R}_{i}E}_{i}{B}_{i}+{\beta\:}_{12}{{R}_{i}B}_{i}H+{\beta\:}_{13}{{E}_{i}B}_{i}H+{\beta\:}_{14}{{R}_{i}E}_{i}H\:+{\beta\:}_{15}{{R}_{i}E}_{i}{B}_{i}H+\:{\beta\:}_{16}{\kappa\:}_{other}\:$$

In the simple model, decision to work *DW* in the trial *i* is predicted by the fixed effects of reward *R*, effort *E*, beneficiary *B*, and their interactions. DW is a binary, factor variable in this logistic mixed-model. The hypocrisy model adds the hypocritical blame index *H* as an independent variable together with its interaction with all the other variables. The harm aversion parameter κ_other_ was also included as a regressor of no interest given that it is strongly correlated with hypocritical blame^9^. For both the simple and the hypocrisy models, random intercepts were clustered in subjects j, and random slopes on effort and reward were included as it is expected that participants vary in their sensitivities to this information. Crucially, AICs for each model were used to test whether the addition of hypocrisy improved the model. This was complemented with a loglikelihood test using the *anova* function in R to test whether differences in model fitting were significant, given that the simple and the hypocrisy models were nested.

For pot-hoc analyses, we used the *emtrends* function in R (*emmeans* package). For these analyses, we took hypocritical blame as a covariate, and compared whether the slope at each level of effort level and reward magnitude combination was different between self and other. Therefore, with this analysis we could test for significant differences of the effects of hypocritical blame on decisions to work between self and other, disentangling the 4-way interaction found in the main results.

To test the influence of hypocritical blame on performance, similar mixed-models to the ones described above were used. In these models, the force exerted in each trial in which participants chose to work were used as dependant variable instead of DW, using the *lmer* function in R. Force was normalised per each trial as a proportion of participant’s maximum to account for between-subject variability in the force exerted and calculated the AUC for the three seconds window in which they exerted force. Thus, normalised force was a continuous variable in these subject-level random-intercept models. Trials where participants chose to rest were excluded from analysis. Unlike the decision models described above, random slopes for effort and reward were not used because (i) participants had different number of trials per each different condition, and (ii) high individual variability in the force exerted between levels of effort and reward was not expected - the force required to obtain the reward on offer was set for each participant’s specific MVC and showed limited variability in existing data^25–27^. Furthermore, interactions between effort x reward x beneficiary were not included, as participants, regardless of hypocritical blame, chose to work for others significantly less often compared to self, especially in low-reward/high-effort trials. This means that a potential 3-way interaction would be unstable because of the small trial sampling at certain combinations of effort and reward.

Finally, the effects of hypocritical blame on performance were also tested with a model that predicted whether each trial was succeeded or failed, i.e. whether people achieved the specific effort requirement for each work trial and therefore whether they obtained the reward on offer. Here, success was a binary factor variable in a logistic random-intercept mixed-model. The success models had the same structure that the force models, i.e. 3-way interactions of effort x reward x beneficiary and random slopes were not included. Notice that even though models predicting success can give important insights about the motivational profile of hypocritical blame, these results must be taken cautiously, as the success rates in this task were very high regardless the condition, and therefore, the effects of hypocritical blame on trial success could be triggered by mainly a few participants/trials.

## Supplementary Information

Below is the link to the electronic supplementary material.

Supplementary Material 1

## Data Availability

All data and scripts used for main analysis and figures can be found here https://osf.io/qmzup/?view_only=b223dbe0b4904c93a64289a695e6ec81.
