# Supplementary material for: Hypocritical blame is associated with reduced prosocial motivation
Source: Sci Rep. 2025 Sep 25;15:32811. doi: 10.1038/s41598-025-17698-4 (PMC12464242; doi:10.1038/s41598-025-17698-4)
Supplement: Supplementary file 1 — Supplementary Material 1 [file 41598_2025_17698_MOESM1_ESM.docx]

**Supplementary Information for “Hypocritical blame is associated with reduced prosocial motivation”**

Luis Sebastian Contreras-Huerta, Hongbo Yu, Annayah M. B. Prosser, Patricia L. Lockwood, Molly J. Crockett and Matthew A.J. Apps

**Correspondence**: Luis Sebastian Contreras-Huerta, sebastian.contreras@uai.cl; Matthew A.J. Apps, [m.a.j.apps@bham.ac.uk](mailto:m.a.j.apps@bham.ac.uk)

**Supplementary Methods**

*Social Manipulation Procedure*

As part of the study, participants had to attend to two experimental sessions. In these sessions, participants made decisions in the harm aversion and the prosocial effort tasks, which count with two types of trials: self trials, where participants themselves were the beneficiaries of the outcomes, and other trials, where outcomes benefited an unknown person. To ensure participants believed in the social aspect of the tasks, we employed a systematic protocol. In this role assignment procedure, participants were informed that two roles existed: the Decider and the Receiver. The Decider would engage in tasks where they could make decisions benefiting themselves or the Receiver, while the Receiver would perform tasks unrelated to benefiting anyone. Participants were led to believe they could be assigned either role through a random selection process involving choosing balls from a box. However, unbeknownst to them, all participants were actually assigned the role of the Decider, while the Receiver was a confederate. After data collection, participants were informed of this deception.

The role assignment process occurred during the first experimental session, and counted with the following stages. Initially, during the preparation phase, participants were situated inside the experimental room alongside the experimenter, who informed the role assignment procedure. Subsequently, random assignment took place when a second experimenter entered the room with the confederate, positioned on opposing sides of the participant behind a partially opened door, ensuring mutual concealment. Both participant and confederate were instructed to refrain from speaking aloud and provided with yellow rubber gloves to obscure identifying features. Once anonymity was confirmed, participants and confederates were prompted to extend their gloved hands towards the door and wave to acknowledge each other's presence. Following this, they proceeded to select a ball from a box held by one of the experimenters, the order determined by a coin toss. Upon completion of the random assignment, the second experimenter and the confederate departed, and participants were notified that they would assume the role of the Decider based on the ball they selected. They were informed that their decisions would remain anonymous, that they would not encounter or interact with the Receiver, and that Receivers would undertake a different unrelated task concurrently. These instructions were designed to alleviate concerns regarding reputation, social desirability, and reciprocity in decision-making, factors known to influence social behaviour.

In the second experimental session, another social manipulation was introduced. Initially, participants were provided with instructions and underwent a calibration procedure for the prosocial effort task. Following this, they were briefed on the main decision task and its social implications. Participants were informed that they would be paired with one of the receivers from the previous experimental session, though not necessarily the same one. A second experimenter then entered the room, and both the confederate/receiver and the participant stood on opposite sides of a partially open door, exchanging waves, akin to the random assignment process in the first experimental session. Subsequently, the second experimenter and the receiver exited, allowing participants to proceed with the prosocial effort task. To validate participants' belief in the social manipulation, subtle debriefing questions were asked after each experimental session, avoiding direct inquiries about the manipulation's credibility. Suspicions or doubts regarding participants' beliefs about the receiver were included as exclusion criteria

*Correlation between decisions in the harm-aversion and prosocial effort tasks*

We conducted a supplementary analysis to test whether decisions that benefit others across task were correlated, which could partly account for the observed effects of hypocritical blame on prosocial effort. Computational models were fitted to both tasks to quantify harm aversion (κ) and effort discounting (λ) parameters, capturing decisional tendencies in the harm-aversion and prosocial effort tasks, respectively. Descriptive statistics are provided in **Supplementary Table S3**.

In the harm-aversion task, κ captures the extent to which participants devalue money when it causes pain to others (see **Materials and Methods**, main text). This parameter was used to calculate the hypocritical blame index.

In the prosocial effort task, effort-based choices were modelled as parabolic discounting of reward by effort:

$$SV=R-\lambda E^{2}$$

$$\lambda=\left\{ \begin{aligned} \lambda_{self} if self trial \\ \lambda_{other} if other trial \end{aligned} \right.$$

Where *SV* is the subjective value of the work offer, *R* the reward magnitude, *E* the effort level, and λ the discounting parameter, indexing how strongly rewards are devalued parabolically by effort. Separate parameters were estimated for self (λ_self_) and other (λ_other_) trials. A softmax function converted subjective values into choice probabilities:

$${P(work)}_{j(i)}= \frac{exp(\beta_{j} {SV}_{j\left( i \right)})}{\exp\left( \beta_{j} 1 \right)+exp(\beta_{j} {SV}_{j\left( i \right)})}$$

Where *P* is the probability to work of a participant *j* in a trial *i.* The probability of resting is given by 1-Pj(i).

We then performed Spearman-rank correlations between prosocial tendencies across tasks, focusing on κ_other and λ_other. For completeness, we also examined κ_self, λ_self, and self–other differences, reported in **Supplementary Table S4.**

**Supplementary Tables**

**Table S1.** Results revealed by the hypocrisy model, where trial-by-trial decisions to work or rest in the prosocial effort task were predicted by the effort level, the reward magnitude, the beneficiary of the reward, and participant’s hypocritical blame index (for details see **Materials and** **Methods** section, **equation 5**, in the main text).

| Effect | beta | SEM | z | p |
| --- | --- | --- | --- | --- |
| Reward | 2.231 | 0.185 | 7.626 | < 0.001 |
| Beneficiary | -3.084 | 0.109 | -28.234 | < 0.001 |
| Effort | -2.496 | 0.214 | -11.684 | < 0.001 |
| Hypocritical Blame | -0.086 | 0.409 | -0.211 | 0.833 |
| Kappa Other | -1.428 | 1.295 | -1.103 | 0.270 |
| Reward*Beneficiary | -0.851 | 0.100 | -8.553 | < 0.001 |
| Reward*Effort | 0.001 | 0.082 | 0.017 | 0.986 |
| Beneficiary*Effort | 0.217 | 0.101 | 2.148 | 0.031 |
| Reward* Hypocritical Blame | -0.034 | 0.184 | -0.182 | 0.855 |
| Beneficiary* Hypocritical Blame | -0.519 | 0.114 | -4.539 | < 0.001 |
| Effort* Hypocritical Blame | -0.182 | 0.213 | -0.856 | 0.392 |
| Reward*Beneficiary*Effort | -0.075 | 0.096 | -0.780 | 0.436 |
| Reward*Beneficiary* Hypocritical Blame | -0.114 | 0.105 | -1.089 | 0.276 |
| Reward*Effort* Hypocritical Blame | -0.162 | 0.084 | -1.939 | 0.053 |
| Beneficiary*Effort* Hypocritical Blame | 0.265 | 0.106 | 2.496 | 0.013 |
| Reward*Beneficiary*Effort* Hypocritical Blame | 0.270 | 0.099 | 2.704 | 0.007 |

**Table S2.** Results revealed by post-hoc analyses on the hypocrisy model unpacking the 4-way interaction found between effort level, reward magnitude, beneficiary and hypocritical blame. In these post-hoc analyses, the effects of hypocritical blame on decisions to work in the prosocial effort task were compared between self and other trials, testing for significance between their slopes in each reward and effort combination.

| *Reward*  *(Credits)* | *Effort Level*  *(%Maximum Voluntary Contraction)* | | | | | | | | | |
| --- | --- | --- | --- | --- | --- | --- | --- | --- | --- | --- |
|  | 30 | | 40 | | 50 | | 60 | | 70 | |
|  | *z ratio* | *p* | *z ratio* | *p* | *z ratio* | *p* | *z ratio* | *p* | *z ratio* | *p* |
| 2 | 0.78 | 0.999 | 1.60 | 0.850 | 2.70 | 0.172 | 2.84 | 0.124 | 2.35 | 0.353 |
| 4 | 2.91 | 0.102 | 3.73 | 0.007 | 4.44 | <0.001 | 3.49 | 0.017 | 2.12 | 0.512 |
| 6 | 3.86 | 0.005 | 4.25 | <0.001 | 4.54 | <0.001 | 3.36 | 0.027 | 1.08 | 0.987 |
| 8 | 3.64 | 0.010 | 3.74 | 0.007 | 3.62 | 0.011 | 2.15 | 0.491 | -0.27 | 1.000 |
| 10 | 3.37 | 0.026 | 3.33 | 0.030 | 2.96 | 0.089 | 1.23 | 0.968 | -0.99 | 0.993 |

**Table S3.** Descriptive statistics (means and standard deviations, SD) for computational parameters in the harm aversion task and the prosocial effort task, which reflect tendencies to avoid harming others and to avoid effort, respectively.

| *Condition* | *Harm Aversion* | | *Prosocial Effort* | |
| --- | --- | --- | --- | --- |
|  | *mean* | *SD* | *mean* | *SD* |
| *Self* | 0.268 | 0.207 | 0.065 | 0.059 |
| *Other* | 0.399 | 0.274 | 0.238 | 0.286 |
| *Self-Other* | -0.131 | 0.267 | 0.173 | 0.293 |

**Table S4.** Spearman correlations (ρ) between decision parameters in the harm aversion and prosocial effort tasks, separated by self trials (where the beneficiary is the participant), other trials (benefiting another person), and their difference. 95% confidence intervals are reported.

| Condition | ρ | p | Lower 95% CI | Upper 95% CI |
| --- | --- | --- | --- | --- |
| Other | -0.097 | 0.455 | -0.341 | 0.158 |
| Self | 0.148 | 0.254 | -0.107 | 0.386 |
| Self-Other | -0.296 | 0.021 | -0.510 | -0.048 |

| Effect | beta | SEM | t | p |
| --- | --- | --- | --- | --- |
| Reward | 0.006 | 0.001 | 5.594 | < 0.001 |
| Beneficiary | -0.016 | 0.002 | -9.927 | < 0.001 |
| Effort | 0.113 | 0.001 | 115.135 | < 0.001 |
| Hypocritical Blame | 0.010 | 0.010 | 0.994 | 0.324 |
| Kappa Other | 0.090 | 0.038 | 2.395 | 0.020 |
| Reward*Beneficiary | 0.002 | 0.002 | 1.394 | 0.163 |
| Beneficiary*Effort | -0.005 | 0.002 | -3.270 | 0.001 |
| Reward* Hypocritical Blame | 0.002 | 0.001 | 1.964 | 0.050 |
| Beneficiary* Hypocritical Blame | -0.006 | 0.002 | -3.941 | < 0.001 |
| Effort* Hypocritical Blame | -0.002 | 0.001 | -1.965 | 0.050 |
| Reward*Beneficiary* Hypocritical Blame | -0.002 | 0.002 | -1.025 | 0.305 |
| Beneficiary*Effort* Hypocritical Blame | 0.001 | 0.002 | 0.687 | 0.492 |

**Table S5.** Results revealed by the hypocrisy model, where force exerted in each trial where participants decided to work in the prosocial effort task were predicted by the effort level, the reward magnitude, the beneficiary of the reward, and participant’s hypocritical blame index (for details see **Materials and** **Methods** section in the main text).

**Table S6.** Results revealed by the hypocrisy model, where trial-by-trial success of achieving the force required when participants decided to work in the prosocial effort task were predicted by the effort level, the reward magnitude, the beneficiary of the reward, and participant’s hypocritical blame index (for details see **Materials and** **Methods** section in the main text).

| Effect | beta | SEM | z | p |
| --- | --- | --- | --- | --- |
| Reward | 0.002 | 0.158 | 0.012 | 0.990 |
| Beneficiary | -0.734 | 0.332 | -2.225 | 0.026 |
| Effort | -1.218 | 0.183 | -6.646 | < 0.001 |
| Hypocritical Blame | 0.059 | 0.339 | 0.174 | 0.861 |
| Kappa Other | 1.360 | 1.012 | 1.344 | 0.179 |
| Reward*Beneficiary | 0.396 | 0.226 | 1.748 | 0.080 |
| Beneficiary*Effort | -0.034 | 0.255 | -0.132 | 0.895 |
| Reward* Hypocritical Blame | 0.175 | 0.147 | 1.191 | 0.234 |
| Beneficiary* Hypocritical Blame | -0.735 | 0.297 | -2.477 | 0.013 |
| Effort* Hypocritical Blame | -0.071 | 0.179 | -0.397 | 0.691 |
| Reward*Beneficiary* Hypocritical Blame | -0.330 | 0.199 | -1.660 | 0.097 |
| Beneficiary*Effort* Hypocritical Blame | 0.133 | 0.236 | 0.562 | 0.574 |

**
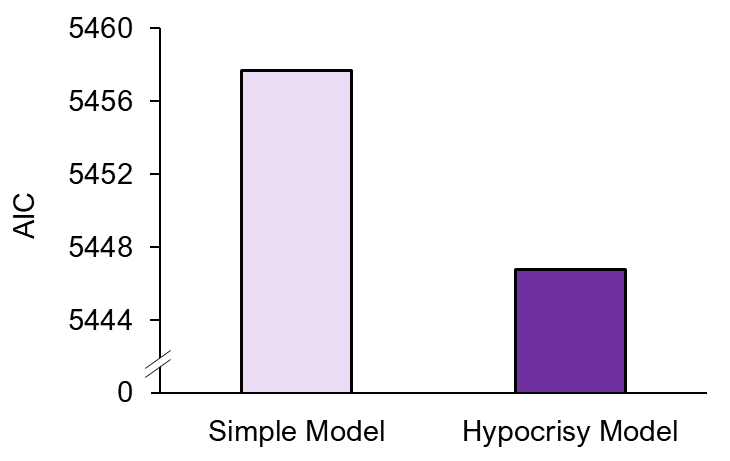
Supplementary Figures**

**Supplementary Figure S1**. Adding hypocritical blame, in addition to effort, reward and beneficiary, as an independent variable into a model predicting decisions to work (**equation 5** in the main text) improves model fitting according to AIC values (Akaike Information Criterion, y-axis)

**Supplementary Figure S2.** *P-values of the contrasts between the effects of hypocrisy on self and other decisions.* In post-hoc analyses, significant differences between the slopes of the effects of hypocrisy on self and other decisions were tested per each combination of reward and effort in the hypocrisy model (**equation 5** in the main text).


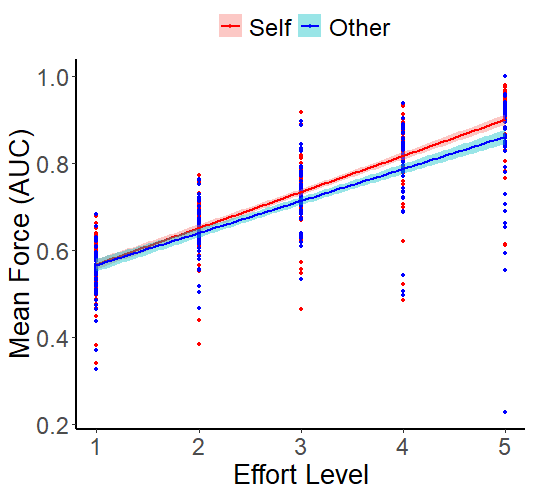


**Supplementary Figure S3.** *The hypocrisy model reveals that people exerted less force when actions benefitted others than self*. Effort and beneficiary interaction indicates that participants exerted less force for others compared to self especially in high effort trials. Y-axis corresponds to the mean area under the curve (AUC) during the 3 seconds force period across effort levels normalised to participants maximum level of force exerted across trials. X-axis corresponds to the different effort levels (from lowest, i.e., 1 = 30% of MVC, to the highest, i.e. 5 = 70% of MVC). Shaded areas show the 90% confidence interval around the slopes. Individual points show the score of each participant for each condition.
